# Preparation of pH-Responsive Tanshinone IIA-Loaded Calcium Alginate Nanoparticles and Their Anticancer Mechanisms

**DOI:** 10.3390/pharmaceutics17010066

**Published:** 2025-01-06

**Authors:** Tianying Ren, Jing Wang, Yingxin Ma, Yichen Huang, Somy Yoon, Lijun Mu, Ru Li, Xuekun Wang, Lina Zhang, Pan Li, Lusha Ji

**Affiliations:** 1 State Key Laboratory for Macromolecule Drugs and Large-Scale Manufacturing, College of Pharmacy, Wenzhou Medical University, Wenzhou 325035, China; dxmei_312@163.com; 2 State Key Laboratory for Macromolecule Drugs and Large-Scale Manufacturing, School of Pharmaceutical Sciences and Food Engineering, Liaocheng University, Liaocheng 252059, China; huangyichen200045@163.com (Y.H.); mlj15215354741@163.com (L.M.); liru0024@163.com (R.L.); xuekunwang0610@126.com (X.W.); 3 College of Pharmacy, Chonnam National University, Gwangju 61186, Republic of Korea; somyyoon@jnu.ac.kr; 4 Key Laboratory for Pediatrics of Integrated Traditional and Western Medicine, Liaocheng People’s Hospital, Liaocheng 252000, China; wangjing20083433@126.com; 5 College of Medicine, Liaocheng Vocational and Technical College, Liaocheng 252000, China; fangyan202109@163.com

**Keywords:** tanshinone IIA, pH sensitive, calcium alginate, autophagy, apoptosis, cancer therapy

## Abstract

**Background:** Tanshinone IIA (Tan IIA) is a lipophilic active constituent derived from the rhizomes and roots of *Salvia miltiorrhiza Bunge* (Danshen), a common Chinese medicinal herb. However, clinical applications of Tan IIA are limited due to its poor solubility in water. **Methods**: To overcome this limitation, we developed a calcium alginate hydrogel (CA) as a hydrophilic carrier for Tan IIA, which significantly improved its solubility. We also prepared nanoparticles with pH-responsive properties to explore their potential for controlled drug delivery. The physicochemical properties of Tan IIA/CA nanoparticles were evaluated, including their size, stability, and release profile. We also utilized RNA sequencing to further investigate the underlying anticancer mechanisms of Tan IIA/CA nanoparticles. **Results**: The Tan IIA/CA nanoparticles demonstrated enhanced solubility and exhibited potent anticancer activity in vitro. Additionally, the nanoparticles showed promising pH-responsive behavior, which is beneficial for controlled release applications. Further investigation into the molecular mechanisms revealed that the anticancer effects of Tan IIA/CA were mediated through apoptosis, ferroptosis, and autophagy pathways. **Conclusions:** This study confirms the anticancer potential and mechanisms of Tan IIA, while also presenting an innovative approach to enhance the solubility of this poorly soluble compound. The use of CA-based nanoparticles could be a valuable strategy for improving the therapeutic efficacy of Tan IIA in cancer treatment.

## 1. Introduction

Cancer currently stands as the predominant cause of mortality in the human population, with its global incidence continuing to rise [1]. Chemotherapy remains the primary therapeutic approach for addressing malignant tumors [2]. Nonetheless, the effectiveness of chemotherapy is constrained by the development of resistance to drugs and the occurrence of severe and undesirable toxic effects [3,4,5]. As a consequence, the identification and development of novel antitumor medications assume critical importance and require immediate attention.

Tanshinone IIA (Tan IIA) is a lipophilic active compound that is derived from the rhizomes and roots of the Chinese medicinal herb *Salvia miltiorrhiza Bunge* (Danshen) [6]. Its pharmacological properties have made it a popular choice for the treatment of cardiovascular diseases [7,8]. Moreover, Tan IIA exhibits a diverse array of biological effects, including anti-inflammatory and anticancer activities [9,10]. In fact, it has been utilized as an antitumor agent against various types of cancer cells, including lung cancer, breast cancer, and glioma [11,12,13].

Natural Tan IIA has been shown to have poor bioavailability due to its low water solubility, first-pass metabolism, and inadequate dissolution rates, which hinder its clinical application [14,15]. The insolubility and insufficient dissolution rates of Tan IIA pose significant challenges. In order to enhance the solubility of Tan IIA, a water-soluble derivative called sodium tanshinone IIA sulphonate (sodium tanshinone IIA sulphonate injection) was developed and is currently being used clinically for the treatment of cardiovascular diseases. However, this structural modification has led to a decrease in the antitumor activities of Tan IIA [16,17]. Therefore, it is necessary and meaningful to explore a technology that does not involve altering the structure of Tan IIA.

Multiple drug carriers have recently been developed to enhance the water solubility of anticancer drugs, including hydrogels [18]. Smart hydrogels are capable of responding to environmental stimuli, such as heat, light, and pH, enabling in situ gelation and controlled drug release [19,20]. This feature significantly improves the convenience and efficiency of drug delivery [21,22]. Alginate, a widely studied polysaccharide, has been extensively investigated for topical drug delivery due to its low toxicity and adjustable properties [22,23]. The hydrophilic polymer has already received approval from the US Food and Drug Administration for biomedical applications [24,25]. Alginate hydrogels can be easily fabricated by crosslinking sodium alginate in a calcium chloride aqueous solution [26]. Additionally, alginate hydrogels offer numerous advantages, including non-immunogenic and biodegradable properties, gel formation under mild conditions, adjustable degradation rate, and biocompatibility [27,28]. These qualities make alginate hydrogels suitable candidates for encapsulating drugs and anticancer agents.

This study describes the preparation of Tan IIA/CA nanoparticles using a modified water-in-oil (w/o) emulsion solvent diffusion method. The loading of Tan IIA onto these nanoparticles was achieved through a simple, low-cost procedure that demonstrated good repeatability and scalability. The Tan IIA/CA nanoparticles exhibited pH-responsiveness characteristics and displayed robust antitumor cell activity based on in vitro cell apoptosis experiments. In addition, the antitumor mechanisms of these nanoparticles were investigated which provides a theoretical basis for the application of TanIIA as an effective anticancer drug.

## 2. Materials and Methods

### 2.1. Materials

Sodium alginate (SA), calcium chloride (CaCl_2_), doxorubicin (DOX), and tanshinone IIA were purchased from Macklin. Dulbecco’s Modified Eagle Medium (DMEM), trypsin, fetal bovine serum (FBS), phosphate-buffered saline (PBS), and penicillin–streptomycin (PS) solution were purchased from Procell (Beijing, China). Polyvinylidene fluoride (PVDF) membranes were purchased from Millipore. RIPA lysis buffer, phenylmethanesulfonyl fluoride (PMSF) (a serine protease inhibitor), protein-loading buffer, biomarkers, and primary antibodies (GPX4, GAPDH, Caspase 3), and secondary antibodies were purchased from Abcam Biotech (Shanghai, China). Cell Counting Kit-8 (CCK-8) was purchased from Dingguo Biology (Beijing, China). HGC 27 cells and GFP+HGC 27 cells were purchased from Zhongqiaoxinzhou Biology (Shanghai, China). HGC27 cells are human gastric cancer cells, which are epithelial in origin. CX-1 cells and GFP+CX-1 cells were purchased from Jingfeng Biology (Shanghai, China). CX-1 cells are human colon carcinoma cells with a morphology typical of colon adenocarcinoma. H293T cells, human embryonic kidney (HEK) cells, were purchased from Cell Bank, Chinese Academy of Sciences. Calcium kit Fluo-3 AM, 2.5% glutaraldehyde and 4% paraformaldehyde were purchased from Yeasen Biotech (Shanghai, China). SDS-PAGE gels, SDS buffer, transfer buffer, enhanced chemiluminescence substrate (ECL) and BCA protein assay kit were purchased from SEVEN Biology (Beijing, China). Trizol reagent was purchased from Sangon Biotech (Shanghai, China). Qubit RNA Assay Kit was purchased from Life Technologies (Carlsbad, CA, USA). Super High-Fidelity Mix Kit was purchased from New England Biolabs (Ipswich, MA, USA). Apoptosis assay kit was purchased from BD Bioscience (San Diego, CA, USA).

### 2.2. Preparation of Calcium Alginate Nanoparticles

The nanoparticles were prepared using a modified water-in-oil emulsification process, as described in the literature [29]. To prepare the nanospheres, a 1 mL solution of 1% sodium alginate was emulsified with sunflower oil containing 2 mL of span 80 for 30 min using an oscillator set at a speed of 2000 rpm. The resulting emulsions were promptly transferred to a mixture of sunflower oil and glacial acetic acid in a 1:1 ratio and stirred at 200 rpm for 15 min at room temperature. In a separate step, 30 mL of a 1 mol/L CaCl_2_ solution was added to the mixture and vigorously agitated for 2 h. Subsequently, the mixtures were centrifuged at 7000 rpm for 10 min. The supernatant was discarded, and the pellet containing the nanospheres was suspended in isopropyl alcohol to remove any remaining sunflower oil. The nanospheres were then washed twice with 10 mL portions of a 1 mol/L CaCl_2_ solution, with each wash being accompanied by gentle vortex mixing for 10 min. Finally, the nanospheres were suspended in a 1 mol/L CaCl_2_ solution and stored at 4 °C.

To prepare the samples loaded with drugs, the nanoparticles were centrifuged at 8000 rpm for 10 min and vacuum-dried for 2 h. Then the nanoparticles were soaked in 4 μM Tanshinone IIA for 1 h and centrifuged again at 8000 rpm for 10 min. The precipitate was collected. The resulting suspension was sonicated at room temperature for 5 min at 20 W using a sonicator. Subsequently, the hydrodynamic size was determined.

### 2.3. Pharmaceutical Characterization

The hydrodynamic size and zeta potential measurements of nanoparticles in distilled water at 25 °C was measured using dynamic light scattering (DLS) with the Malvern Zetasizer Nano. DLS measured the size of CA and Tan/IIA nanoparticles at predetermined time points (0 day, 7 day or 14 day). Nanoparticle sizes were quantified by transmission electron microscopy (TEM).

#### Encapsulation Efficiency and Loading Efficiency

For the treatment of Dox and Tan IIA samples, 1 mL of double distilled water (ddH2O) was combined with the tested sample. The drug loaded nanoparticles were prepared using a similar method referred to in Section 2.2. A serial dilution of Tan IIA was chosen as a standard curve and total Tan IIA content in the sample was calculated using the standard curve. Method of measurement of concentration of nanoparticles loaded with DOX was the same as mentioned above. The resulting solution was then subjected to analysis using a Nanodrop 2000 spectrophotometer. The drug concentration in the solution was measured using HPLC at a wavelength of 268 nm for TanIIA and 480 nm for DOX [30].

Drug loading content (%) = (weight of loaded drug (DOX or Tan IIA))/total weight of nanoparticles × 100 Encapsulation efficiency (%) = (weight of loaded drug/weight of total drug) × 100

### 2.4. Cell Culture

The CX 1 and HGC 27 cell lines were obtained from the Cell Bank of the Chinese Academy of Sciences and subjected to STR DNA profiling. The cells were grown in DMEM medium supplemented with 10% FBS and 1% PS and maintained in a 37 °C incubator with 5% CO_2_.

### 2.5. CCK 8 Assay

Cells were seeded into 96-well plates at a density of 1 × 10^5^ cells/mL and cultured in 100 μL of DMEM medium for 24 h. Subsequently, nanoparticles were introduced into the plates and incubated for 24 h, 48 h, 72 h, and 96 h, respectively, followed by the addition of 10 μL CCK-8 solution. The optical density (OD) at 450 nm was measured after a 2-h incubation period at 37 °C.

### 2.6. Flow Cytometry Analysis

Cells were seeded into 6-well plates at a density of 1 × 10^6^ cells/mL, and nanoparticles (final conc. 4 µM) were added into cells for 24 h. Afterward, cells were washed with PBS and collected by trypsin treatment. Subsequently, these cells were stained with the 7-AAD Annexin V apoptosis detection kit (BD Bioscience) following the manufacturer’s instructions and analyzed using a BD flow cytometry (BD Arial III system). Each experiment was performed in triplicate. For these analyses, a total of 10,000 cells were acquired and further analyzed using FlowJo software version10. The Annexin V-positive and double Annexin V/7AAD-positive cells were considered apoptotic.

### 2.7. Western Blots Assay

For the western blot assay, cells were seeded into 6 cm cell dishes at a density of 1 × 10^5^ cells/mL for 24 h. The cells were then washed with PBS three times and lysed using RIPA buffer and protease inhibitors. The protein concentration was determined using a BCA protein assay kit. An equal amount of total protein was loaded onto SDS-PAGE gels and subsequently transferred to a PVDF membrane. The PVDF membrane was blocked using quick blotting buffer for 10 min and washed three times with TBST buffer. Next, the PVDF membrane was incubated with the primary antibody, followed by a secondary antibody conjugated with horseradish peroxidase. The blots were visualized using the Bio-rad ChemiDoc Imager. All western blots were performed in triplicate as independent experiments, with GAPDH chosen as the internal reference.

### 2.8. Cell Migration Assay

Migration ability was assessed using a scratch migration assay in which 5 × 10^4^ cells/mL were seeded in 3.5 cm cell dishes and incubated for 24 h. After that, a vertical scratch was created using a 200 µL pipette tip. The scratch zones were photographed and measured at 0 h and 24 h (Olympus, Japan). The cell scratch area was measured using Image J software 1.45. The extent to which the wound had closed over 24 h was calculated and expressed as a percentage of the difference between time points 0 and 24 h. Each condition was tested three times.

### 2.9. Measurement of Intracellular Ca^2+^ Level

Cells were seeded in 12-well plates at a density of 1 × 10^5^ cells/mL and the cells were cultured for 24 h. Then the cells were treated with TanIIA/CA nanoparticles for 12 h. Subsequently, the cells were collected and treated with 5 mM of fluo-3 AM in order to monitor the alterations in intracellular Ca^2+^ levels. The changes were observed using an inverted confocal laser scanning microscopy. Furthermore, the fluorescent intensity of the stained cells was assessed through flow cytometry (BD Arial III), utilizing an excitation wavelength of 488 nm.

### 2.10. Confocal Imaging of Cells

Cells were seeded in 6-well plates at a density of 1 × 10^5^ cells/mL and incubated for 24 h. The nanoparticles carrying Tan IIA (final concentration 4 µM) were then added to the cells for a 2-h period. Following this, the cells were fixed in 4% paraformaldehyde. Confocal images of the cells were obtained using a Leica laser scanning confocal microscope (Leica SP5 spectral laser scanning confocal microscope, Wetzlar, Germany).

### 2.11. Fourier Transform Infrared (FTIR)

The absorption spectra were evaluated using a Varian 3000 Fourier Transform Infrared (FTIR) spectrophotometer. The FTIR technique was employed to characterize the chemical properties of the calcium alginate hydrogel. Peak shifting suggested TanIIA was successfully incorporated into calcium alginate nanoparticles.

### 2.12. Transmission Electron Microscopy (TEM) and Scanning Electron Microscopy (SEM)

Cell suspension was prepared by digesting cells with trypsin, followed by fixation in 2.5% glutaraldehyde for a minimum of 12 h. Subsequently, an examination of the cells was conducted using a JEM-1400 transmission electron microscope. SEM analysis was conducted on a field-emission SEM (Hitachi SU8000 FE-SEM) at 5 kV.

### 2.13. HPLC Analysis to Measure the TanIIA Contents

The chromatographic conditions were as follows: flow rate 1 mL/min, column temperature of 25 °C, sample loading volume of 10 mL. The absorbance was at 268 nm, and the mobile phases were 0.026% phosphoric acid aqueous solution and acetonitrile, respectively, with gradient elution.

The standard curve was made by plotting the peak area from five gradient concentrations. By plotting the peak area (y) of the analyte and the corresponding concentration (x, mg/mL), the linear relationship of each standard curve was determined. The regression equation and correlation coefficient are y = 0.0006x + 0.0068 (R^2^ = 0.9998).

### 2.14. Solubility Determination

The solubility of Tanshinone IIA, Tan IIA/CA, was assessed by adding a measured quantity of each compound to test tubes containing 5 mL of degassed water. The test tubes were sealed and shaken at 25 °C for 24 h. Afterward, the mixtures were centrifuged at 10,000 rpm for 15 min, and the supernatant was obtained. The concentration of each compound in the supernatant was then quantified using high-performance liquid chromatography (HPLC).

### 2.15. Statistical Analysis

The experiments were conducted in three independent replicates. The data were presented as a mean ± standard error. Student’s *t*-test was used to compare whether significance existed between the control and experimental groups based on one factor; *p* values < 0.05 were considered statistically significant. All data was measured via GraphPad Prism (version 7.03).

### 2.16. RNA Extraction and Transcriptome Sequencing

Transcriptome sequencing was performed by Sangon Biotech Corporation (Shanghai, China). Briefly, total RNA was extracted from the frozen samples of two replicates using Trizol reagent. RNA integrity and pollution were detected on 1% agarose gel electrophoresis. RNA concentration was measured using Qubit RNA Assay Kit (Life Technologies). mRNA was enriched and purified from the total RNA using mRNA Capture Beads, and then broken into short fragments. The first- and second-strand cDNA fragments were synthesized after reverse transcription reaction. The double-stranded cDNAs were enriched and purified by Hieff NGS™ DNA Selection Beads. The double-stranded cDNAs were used for end reparation, poly (A) addition, and ligation with sequencing DNA adaptors. Following purification and size selection, cDNA libraries were generated from appropriate size of DNA fragments using Super Canace™ High-Fidelity Mix Kit. The quality of cDNA libraries was detected Qubit DNA Assay Kit. Finally, the cDNA libraries were sequenced on an Illumina NovaSeq6000 platform.

### 2.17. Sequence Assembly, Functional Annotation and Classification

Base calling was transferred to sequenced reads (i.e., raw reads). The quality of sequencing data was evaluated by checking the distributions of the sequencing error rate and A/T/G/C content. Meanwhile, reads with an adapter, reads with ‘N’ base, low quality reads with a Qphred ≤20, length less than 35 nt, and their paired reads were removed. The remaining reads were clean reads. Transcriptome assembly was performed using Trinity software v2.1.1. Gene function annotation was analyzed by BLASTX alignment against the NR (NCBI non-redundant protein database), KEGG (Kyoto encyclopedia of genes and genomes), Pfam, and GO (Gene Ontology) databases (E-value ≤ 1^−5^). The gene expression levels were estimated with TPM (Transcripts Per Million). The *p*-value < 0.05 and the log2 (Treat/Control) > 1.5 were used as the threshold to discriminate the DEGs (differential gene expression).

## 3. Results

### 3.1. Characterization of Nanoparticles

**Figure 1:**
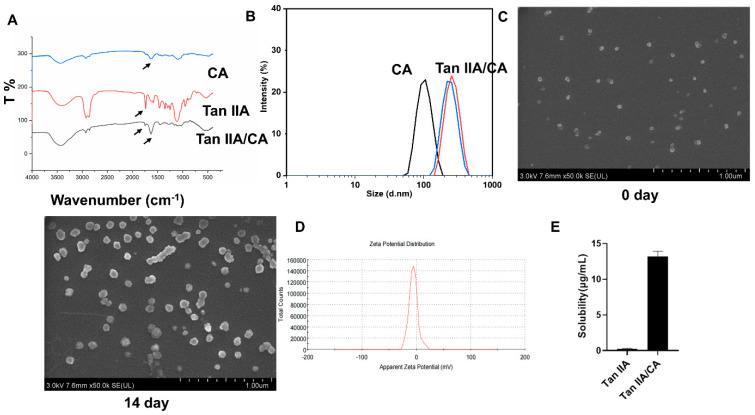
Characterization of nanoparticles. (**A**–**D**) the Fourier transform infrared (FTIR) spectra, the hydrodynamic particle size (nm), scanning electron microscopy (SEM) images, and zeta potential of Tan IIA/CA nanoparticles, respectively. (**A**) Peak shifting suggest TanIIA was successfully incorporated into calcium alginate nanoparticles. The arrows indicate C=O symmetrical and asymmetrical vibrations. (**B**) The changes in particle size meant the TanIIA was successfully incorporated into calcium alginate nanoparticles. (**C**) SEM images between day 0 and day 14 suggest the stability of nanoparticles loaded with TanIIA. (**D**) Zeta potential of nanoparticles loaded with TanIIA. (**E**) The solubility of TanIIA and TanIIA/CA presented as the mean ± SD.

In order to characterize interactions between the drugs (Tan IIA) and carriers, the FTIR spectrum of pure Tan IIA, Tan IIA/CA, and CA hydro polymers were investigated (Figure 1A). The FTIR spectrum of Tan IIA/CA was the superposition of spectra of Tan IIA and CA nanoparticle. Analysis of the spectra for either Tan IIA/CA or individual components did not show any changes for the specific absorption band for the polymers or the Tan IIA, demonstrating that there were no chemical interactions between Tan IIA and CA nanoparticles. Peak shifting suggests TanIIA was successfully incorporated into calcium alginate nanoparticles. The arrows indicate C=O symmetrical and asymmetrical vibrations.

Tan IIA/CA particles were synthesized by the modified W/O emulsion cation method and the size of the nanoparticles was characterized by DLS analysis (Figure 1B). Hydrated nanoparticles displayed a larger particle size due to water absorption and swelling, with size ranging from 100 to 400 nm. SEM image of these nanoparticles is shown in Figure 1C. SEM results revealed the nanoparticles were well-dispersed and with good stability (within 14 days). Zeta potential of the nanoparticles was about −8.41 mV, which indicated that these particles showed a small amount of surface charge (Figure 1D). The solubility of the Tan IIA/CA was found to increase significantly compared to the pure drug (Figure 1E).

### 3.2. Drug Encapsulation and Release In Vitro

To evaluate the efficacy of CA hydrogel carriers, an analysis of drug encapsulation and release rates was conducted. The drug encapsulation rate was 57.8 ± 5.6% and 46.5 ± 3.2%, respectively, for DOX and Tan IIA while the loading capacities were 50.2 ± 5.1% and 42.4 ± 4.5% (Table 1).

**Figure 2:**
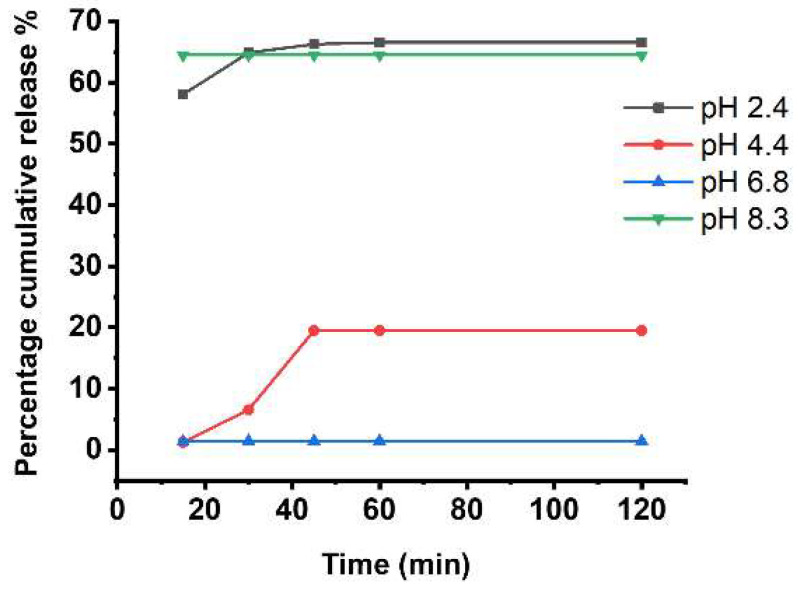
Cumulative drug release percentage in various pH solutions.

Furthermore, the release patterns of Tan IIA/CA to investigate the drug release behavior in CA hydrogels were assessed. The cumulative release amount at pH 2.4 and pH 8.3 exceeded 60% (Figure 2). Notably, a rapid release occurred within 30 min at pH 2.4 and pH 8.3, indicating that changes in pH can trigger the release of Tan IIA. Conversely, the release amount was less than 20% at pH 4.4 and pH 7.8 (Figure 2). These findings collectively demonstrate the remarkable pH-responsiveness characteristics of CA hydrogels.

### 3.3. Nontoxic Properties

**Figure 3:**
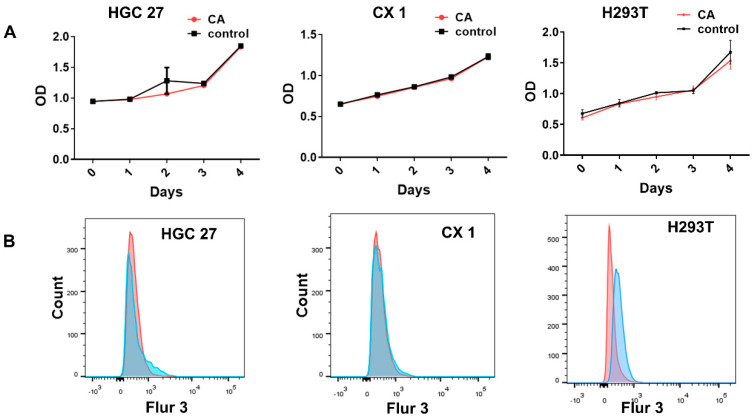
Non biological toxicity of CA particles measured by CCK 8 assay and calcium concentration assay on cells. (**A**) CCK 8 assay of cells after being treated with CA particles for 4 days. (**B**) Flow cytometry assay of intracellular calcium ion (Ca^2+^) concentration of cells after being treated with CA particles. Data are shown as Mean ± SD. Experiments were performed in triplicate.

To confirm the non-toxicity of the hydrogel, CCK-8 assays were conducted to assess cell proliferation using HGC27, CX1, and H293T. Cells were cultured in the presence and absence of CA hydrogels for a duration of 4 days. The results of the CCK-8 assays indicated that there was no substantial variance in cell viability between the experimental and control groups (Figure 3A). To analyze whether calcium alginate microspheres may cause changes in intracellular calcium ion concentration, Ca^2+^ concentration was analyzed by Flow cytometry. The cells were treated with TanIIA/CA nanoparticles for 12 h. Subsequently, the cells were collected and stained with a Fluo-3 AM probe. There was no significant increase observed in free intracellular calcium concentrations (*p* > 0.05) (Figure 3B). Together, these findings demonstrate the biocompatibility of the CA nanocarriers with the tested cells.

#### Drug Uptake Ability In Vitro

**Figure 4:**
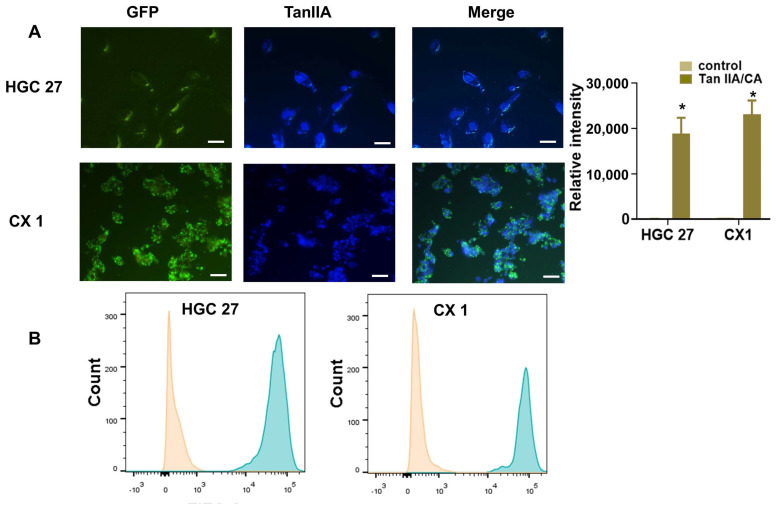
Cellular uptake were shown by confocal laser microscopy and flow cytometry in cells treated with Tan IIA/CA particles. Quantification of fluorescent intensity measured by Image J software. Data are shown as Mean ± SD. Experiments were performed in triplicate, * *p* < 0.05. (**A**) The fluorescence confocal image images of particles size and distribution in cells (unit: μm). Scale bar: 20 μm. (**B**) Flow cytometry analysis of cells treated without or with Tan IIA/CA particles. GFP means green fluorescent protein.

To investigate the cellular uptake capability of CA nanoparticles, Tan IIA/CA was employed as the targeted drug, owing to its intrinsic fluorescence of Tan IIA. As depicted in Figure 4A, the drug was observed to be rapidly internalized by the cells. Furthermore, the fluorescence intensity exhibited by the Tan IIA/CA groups was found to be significantly greater when compared to the control groups (Figure 4A). In this experiment the controlled groups referred to cells with blank CA nanoparticles.

The fluorescence signals in cellular samples were also analyzed using flow cytometry; the corresponding results are presented in Figure 4B. The figure clearly illustrates that the fluorescence intensity observed in the Tan IIA/CA groups is significantly stronger than that observed in the control groups. These findings are in line with the results obtained from the confocal microscope analysis.

**Figure 5:**
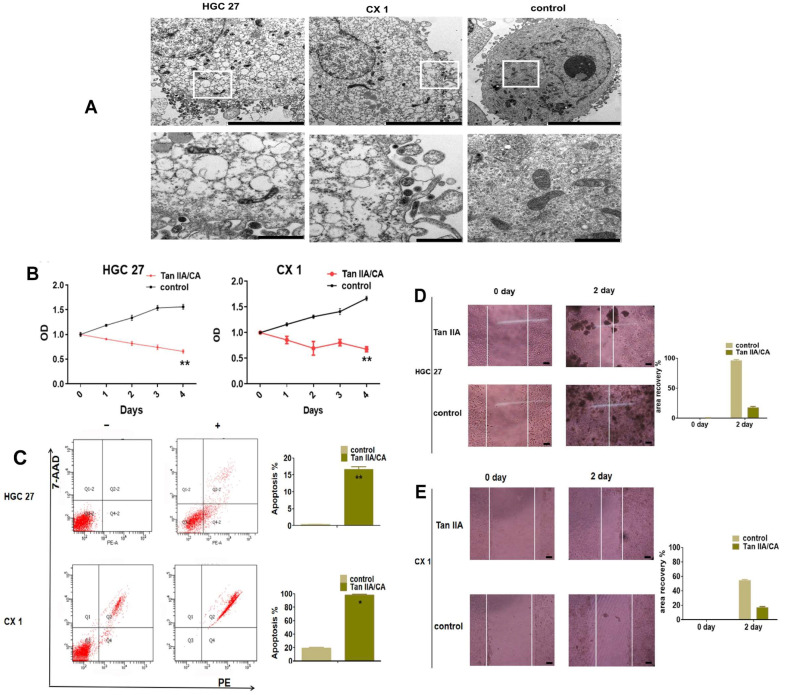
Comparisons of apoptosis levels in cells treated with Tan IIA/CA and control group. (**A**) The TEM images of particles size and distribution in cells (unit: 500 nm). (**B**) Apoptosis was measured with CCK 8 kit. Quantification of apoptosis percentage are shown as Mean ± SD, ** *p* < 0.01. (**C**) Analysis of frequency of cell apoptosis using flow cytometry. Quantification of apoptosis percentage is shown as Mean ± SD. Experiments were performed in triplicate, * *p* < 0.05, ** *p* < 0.01. (**D**,**E**) HGC 27 and CX 1 cells treated with Tan IIA/CA particles and control and cell migration ability assessed with cell scratch assay. The image were taken at day 0 and day 2 after the gap were scratched. Quantification of the relative recovery area is measured by Image J software. Data are shown as Mean ± SD. Experiments were performed in triplicate, * *p* < 0.05, ** *p* < 0.01.

To confirm the uptake of drugs by cells, a transmission electron microscope (TEM) analysis, as depicted in Figure 5A, was conducted. In this particular experiment, Tan IIA/CA nanoparticles were utilized as our experimental groups. The nanoparticles were observed within the plasma of the cells treated with Tan IIA/CA nanoparticles. Consequently, these findings collectively indicate that the uptake of CA nanoparticles by cells is facilitated.

### 3.4. Anticancer Ability In Vitro

The cell killing ability of Tan IIA/CA nanoparticles was assessed in vitro using CCK8 assays and flow cytometry assays. The CCK8 assays demonstrated a significant decrease in cell proliferation ability in the groups treated with Tan IIA/CA (Figure 5B). Flow cytometry analysis of cell apoptosis revealed a significant increase in cell apoptosis in the groups treated with Tan IIA/CA nanoparticles compared to the control groups (Figure 5C). Transmission electron microscope (TEM) was performed, as shown in Figure 5A, which revealed the presence of nanoparticles in the plasma of cells treated with Tan IIA/CA nanoparticles. These cells treated with Tan IIA/CA nanoparticles exhibited altered morphology, showing shrinkage and cell membrane rupture. Furthermore, cell scratch assay showed that Tan IIA/CA decreased cell migration significantly after 48-h incubation (Figure 5D,E). These results demonstrate that the Tan IIA/CA exert effectively anti-tumor activity.

### 3.5. Transcriptomic Analyses

To find the mechanisms of anticancer effects induced by Tan IIA/CA, transcriptomic analyses were performed. Eight cDNA libraries were constructed from the control group (H1, H2, C1, and C2) and Tan IIA treatment group (HT1, HT2, CT1, and CT2), respectively (Table 2). After removing adaptor sequences and low-quality and ambiguous “N” sequences, transcriptome sequencing generated 214,801,546 clean reads and 3.1 × 10^10^ bp in group HT vs. H of HGC27 cancer cells with more than 51,000,000 reads and 7.4 × 10^9^ bp per sample. Meanwhile, a total of 200,076,854 reads and 2.8 × 10^10^ bp was acquired from group CT vs. C of CX1 cancer cells, with more than 44,000,000 reads and 6.4 × 10^9^ bp per sample. Average reads were at least 144 bp in length per sample. The Q20 values of reads were greater than 98.8%. Q30 scores were over 96.2% in all samples. The contents of GC ranged from 52.6% to 54.4%. These values indicated a high quality of clean reads.

### 3.6. Differentially Expressed Genes (DEGs)

**Figure 6:**
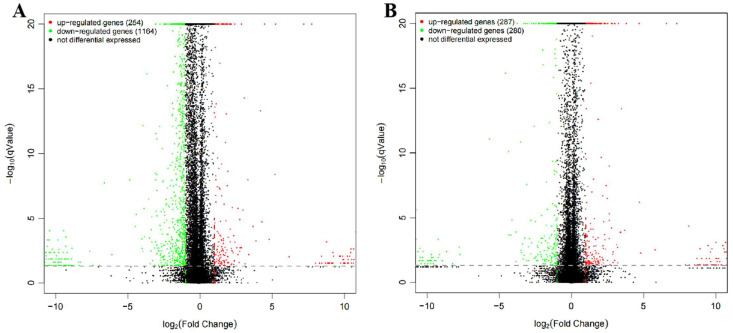
Volcano plot displaying the DEGs. (**A**) Volcano plot of DEGs in group HT vs. H of HGC 27 cancer cells. (**B**) Volcano plot of DEGs in group CT vs. C of CX 1 cancer cells. The *X*-axis indicates fold-change of gene expression (threshold, |log2 (Treat/Control)| > 1), while the *Y*-axis means the statistically significant level (threshold, *p*-value < 0.005). Red dots indicate groups of upregulated genes, green dots indicate groups of downregulated genes, and black dots indicate non-significantly differentially expressed genes (DEGs). Abbreviations: CT: CX1 cells treated with Tan IIA/CA; HT: HGC 27 cells treated with Tan IIA/CA.

To compare the DEGs in group HT vs. H of HGC27 cancer cells and group CT vs. C of CX1 cancer cells, DEGs with volcano plots were identified (Figure 6). In all, 1418 genes were identified as DEGs in group HT vs. H, of which 254 DEGs were significantly upregulated and 1164 DEGs were significantly downregulated (Figure 6A). According to the results of transcriptome analysis, we obtained 567 DEGs in group CT vs. C, of which 287 were up-regulated and 280 were down-regulated (Figure 6B).

### 3.7. Gene Ontology (GO) Analyses and KEGG Pathway Analyses of DEGs

**Figure 7:**
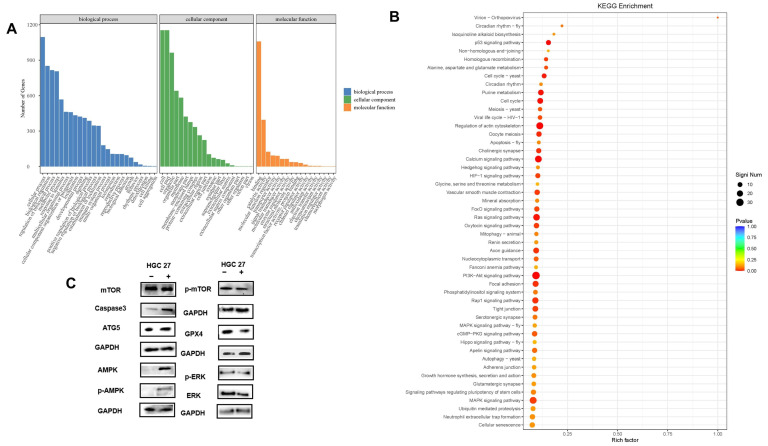
Functional enrichment analysis of DEGs in HGC 27 treated with or without Tan IIA/CA. (**A**) The classification of Gene Ontology in group HT vs. H of HGC 27 cancer cells. (**B**) KEGG pathway enrichment in group HT vs. H of HGC 27 cancer cells. Rich factor represents the ratio of the number of DEGs to the total number of annotated genes in this pathway. Abbreviations: + : HGC 27 cells treated with Tan IIA/CA; −: HGC 27 cells without treated with Tan IIA/CA. (**C**) Western blots were performed to evaluate the expression levels of ATG 5, GPX4, Caspase 3, AMPK, mTOR, ERK, and p-ERK in HGC 27 cells following Tan IIA/CA treatment, with GAPDH serving as the control. Experiments were performed in triplicate. Original blots/gels are presented in the supplementary document.

To explore the biological functions of the DEGs, GO (Gene Ontology) functional annotation and KEGG (Kyoto Encyclopedia of Genes and Genomes) enrichment analysis was conducted. First, GO functional annotation of DEGs in group HT vs. H of HGC27 cancer cells were performed. The DEGs were classified into three main categories and further divided into 62 functional subgroups in group HT vs. H (Figure 7A). In biological process (BP), these genes were predominantly distributed into cellular process (GO:0009987), biological regulation (GO:0065007), regulation of biological process (GO:0050789), and metabolic process (GO:0008152). In cellular component (CC), cell (GO:0005623) and cell part (GO:0044464) are significantly enriched GO terms, followed by organelle (GO:0043226). In molecular function (MF), the majority of DEGs were involved in binding (GO:0005488) and catalytic activity (GO:0003824). For KEGG pathway enrichment analysis, DEGs of group HT vs. H were significantly enriched in 24 pathways, which are related to apoptosis, autophagy, cellular senescence, ferroptosis, and immune system (Figure 7B). Of these, the PI3K-Akt signaling pathway (ko04151), regulation of actin cytoskeleton (ko04810), the MAPK signaling pathway (ko04010), the calcium signaling pathway (ko04020), the Ras signaling pathway (ko04014), the Rap1 signaling pathway (ko04015), focal adhesion (ko04510), cell cycle (ko04110), and the cGMP-PKG signaling pathway (ko04022) were the highly enriched pathways.

**Figure 8:**
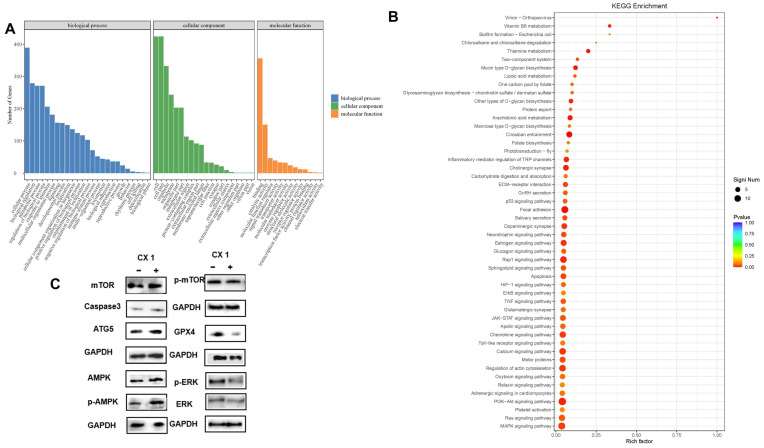
Functional enrichment analysis of DEGs in CX1 treated with or without Tan IIA/CA. (**A**) The classification of Gene Ontology in group CT vs. C of CX1 cancer cells. (**B**) KEGG pathway enrichment in group CT vs. C of CX1 cancer cells. Rich factor represents the ratio of the number of DEGs to the total number of annotated genes in this pathway. Abbreviations: + means CX1 cells treated with Tan IIA/CA. (**C**) Western blots were performed to evaluate the expression levels of ATG 5, GPX4, Caspase 3, AMPK, mTOR in CX1 cells following Tan IIA/CA treatment, with GAPDH serving as the control. Experiments were performed in triplicate. Original blots/gels are presented in the supplementary document.

Subsequently, GO functional annotation and KEGG pathway enrichment analysis were conducted to characterize the roles of the DEGs in group CT vs. C of CX1 cancer cells. The DEGs were enriched with 58 functional subgroups in group CT vs. C, including 25 GO terms in the BP category, 20 GO terms in the CC category, and 13 terms in the MF category (Figure 8A). In the BP category, DEGs were mainly annotated in cellular process (GO:0009987), biological regulation (GO:0065007), metabolic process (GO:0008152), regulation of biological process (GO:0050789), and response to stimulus (GO:0050896). In the CC category, these unigenes were predominantly distributed into cell (GO:0005623) and cell part (GO:0044464), organelle (GO:0043226), and membrane (GO:0016020). In the MF category, binding (GO:0005488) and catalytic activity (GO:0003824) had the largest numbers of DEGs. For the KEGG pathway enrichment analysis, we found that the DEGs of group CT vs. C were mainly classified into 32 pathways, which are related to immune system, autophagy, ferroptosis, and apoptosis (Figure 8B). The significant pathways were the PI3K-Akt signaling pathway (ko04151), the MAPK signaling pathway (ko04010), focal adhesion (ko04510), the calcium signaling pathway (ko04020), regulation of actin cytoskeleton (ko04810), the Rap1 signaling pathway (ko04015), circadian entrainment (ko04713), the Ras signaling pathway (ko04014), the JAK-STAT signaling pathway (ko04630), the TNF signaling pathway (ko04668), glutamatergic synapse (ko04724), and the chemokine signaling pathway (ko04062).

To further verify these results, WB in seven differentially expressed proteins (GPX4, Caspase 3, ATG 5, AMPK, phosphorylated AMPK, mTOR phosphorylated mTOR, ERK, and phosphorylated ERK) were performed (Figure 7C and Figure 8C). GPX4 is a key protein to inhibit ferroptosis; a decline in GPX4 induces ferroptosis. Caspase 3 plays a key role in induced apoptosis and ATG 5 is a key protein involved in autophagy. Moreover, the AMPK/mTOR signaling pathway is a key signaling pathway involved in autophagy. WB results showed increased levels of ATG 5 and Caspase 3, and decreased levels of GPX4, AMPK, phosphorylated AMPK, mTOR, and phosphorylated mTOR in both HGC 27 and CX 1 cells treated with Tan IIA/CA. These results also reveal that Tan IIA/CA perform anti-tumor functions through promoting apoptosis, autophagy, and ferroptosis. These results are consistent with those results of second generation sequencing.

## 4. Discussion

**Figure 9:**
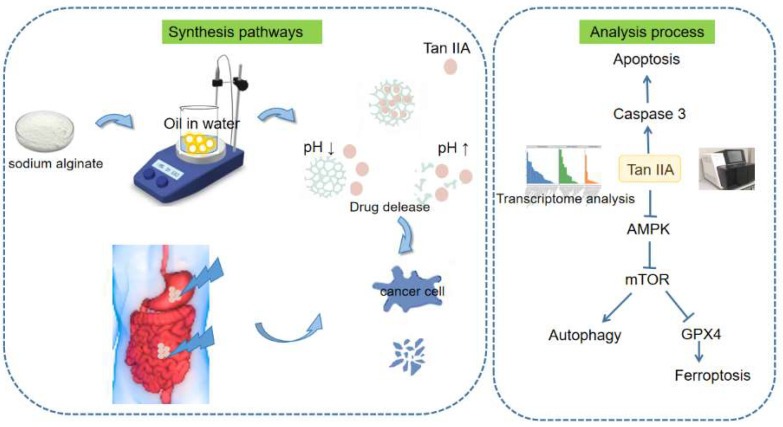
Schematic illustration depicting the fabrication process of Tan IIA/CA particles.

Tan IIA is an important fat-soluble compositions of *Salvia miltiorrhiza*, which has been widely used for treating various cardiovascular diseases and also has antitumor potential. Poorly water-soluble drugs are a common obstacle in pharmaceutical development, presenting a significant challenge. The limited solubility of these drugs often leads to suboptimal bioavailability and reduced therapeutic effectiveness. Consequently, increasing efforts have been dedicated to improving the solubility of poorly water-soluble drugs. This is performed to enhance their overall performance and optimize drug delivery, ultimately resulting in improved therapeutic outcomes. The CA nanoparticles were synthesized using an improved method [29]. As illustrated in Figure 9, a Tan IIA/CA drug delivery system was prepared. Nanoparticles are manufactured by W/O emulsification technique. To improve loading capacity of Tan IIA/CA, we first synthesized drug free nanoparticles (CA) and then encapsulation of Tan IIA was carried out by mixing Tan IIA with CA nanoparticles. In this study, we compare the solubility of Tan IIA/CA and pure drug. We found that the nanoparticles increased the solubility of Tan IIA, highlighting the enhanced therapeutic effects of the nanoparticles. On the other hand, as previous studies have shown, CA hydrogels have characteristics of pH responsiveness and biocompatibility [31]. In alkaline conditions, calcium alginate nanoparticles showed drug release ability due to their simultaneous swelling and degradation [32,33]. In pH 2 conditions, calcium alginate nanoparticles exhibited drug release ability due to matrix shrinkage [32,34]. Tan IIA/CA in this research also displays the same characteristics, a response to dual pH. With the ability to respond to changes in pH, Tan IIA/CA can therefore facilitate accurate and controlled release of drugs.

Since nanoparticles have pH responsiveness, human gastric cancer cell line (HGC 27) and colorectal cancer cell line (CX1) were selected as the research objects. Colon pH is alkaline (pH 8.3–8.4) and gastric pH is acidic [35,36]. In vitro experiments demonstrated that Tan IIA exerts its anti-tumor activity against HGC 27 and CX1 cancer cells. This is consistent with previous studies [37]. However, the mechanisms of Tan IIA antitumor activity remain unclear. However, this research also has a limitation, a low encapsulation rate. To make drug encapsulation rates up to 70–80% is a large challenge, especially for poorly soluble drugs. Our method has increased the drug encapsulation rates compared with other similar nanoparticles [29]. Maybe the ratio between calcium chloride and drugs could influence the encapsulation efficiency. This is a question which needs to be researched in more detail in the future.

Concerned that CA nanoparticles contained Ca^2+^, we analyzed intracellular calcium concentrations with flow cytometry. There was no significant change in levels of Ca^2+^ ions. These results proved that the anticancer effects of Tan IIA/CA are not correlated with CA carriers. This is consistent with our RNA-seq results.

In in vitro experiments, Tan IIA/CA significantly inhibited cell proliferation and reduced the migration of HGC 27 and CX1 cells. The mechanistic study revealed that the anticancer effect of Tan IIA/CA can exert anti-tumor effects by regulating ferroptosis and apoptosis. These results were also verified at the protein level by WB. Increased expression of Caspase 3 and decreased expression of GPX4 proteins were observed. The AMPK/mTOR signal pathways are key signaling pathways involved in regulating autophagy [38]. AMPK is verified to be a promoter of autophagy, and it inhibits mTOR activity to promote autophagy in cancer progression [39]. Ferroptosis is believed to be regulated by the AMPK signaling pathway, and activation of AMPK/mTOR inhibits ferroptosis [40]. In our research, Tan IIA/CA also induced autophagy through the AMPK/mTOR signal pathway in the same way. Although we screened the molecular mechanisms of Tan IIA/CA induced antitumor and some signaling pathway were confirmed in this study, these underlying antitumor mechanisms of Tan IIA/CA are poorly understood. In this study, we analyzed significantly changing gene functions and the corresponding pathways with GO and KEGG enrichment analyses. Our data showed that the most significantly involved pathways were apoptosis, autophagy, cellular senescence, ferroptosis, and immune system. Our data verified the involvement of apoptosis, autophagy, and ferroptosis in the antitumor mechanisms of Tan IIA/CA. Molecular details of the immune system will need further elaborations.

Taken together, our findings suggest that Tan IIA can suppress cancer cell growth, migration, and invasion as well as promote cell apoptosis. Our findings provide a theoretical basis for using Tan IIA as an antitumor drug and providing a strategy for the higher efficiency use of poor water solubility drugs.

## 5. Conclusions

This study revealed that calcium alginate hydrogel is a strategy to improve the insolubility of Tanshinone IIA. Furthermore, we observed that calcium alginate hydrogel has good characteristics of pH-responsiveness, biocompatibility, and nontoxicity. Our findings not only establish an effective approach but elucidate the molecular mechanism underlying Tanshinone IIA induced antitumor effects. Tanshinone IIA can exert antitumor effects by promoting cell apoptosis, autophagy, ferroptosis, and immunology. These results provide a scientific foundation for designing the controlled release of anticancer drugs and the application of Tanshinone IIA as cancer drugs. Tan IIA seems to be an effective antitumor drug.

## Figures and Tables

**Table 1:** The encapsulation efficiency and loading capacity of DOX/CA and Tan IIA/CA particles.

Particles	Encapsulation Efficiency %	Loading Capacity %
DOX/CA	57.8 ± 5.6	46.5 ± 3.2
Tan IIA/CA	50.2 ± 5.1	42.4 ± 4.5

**Table 2:** Summary of RNA-seq clean data in group HT vs. H of HGC27 cancer cells and group CT vs. C of CX1 cancer cells. Abbreviations: CT: CX1 cells treated with Tan IIA/CA; HT: HGC 27 cells treated with Tan IIA/CA.

**Sample**	**HT1**	**HT2**	**HT3**	**H1**	**H2**	**H3**
Total Reads Count (#)	55,590,646	51,585,078	55,087,862	51,282,474	56,343,348	53,812,911
Total Bases Count (bp)	8,018,322,967	7,455,267,812	773,679,538	7,466,638,761	8,170,208,380	7,818,423,571
Average Read Length (bp)	144.24	144.52	144.45	145.6	145.01	145.3
Q10 Bases Count (bp)	8,001,423,705	7,439,545,492	772,048,462	7,449,007,281	8,151,318,468	7,800,162,875
Q10 Bases Ratio (%)	99.79%	99.79%	99.7%	99.76%	99.77%	99.7%
Q20 Bases Count (bp)	7,952,826,783	7,394,373,331	767,360,026	7,397,235,782	8,096,391,117	7,746,813,450
Q20 Bases Ratio (%)	99.18%	99.18%	99.1%	99.07%	99.10%	99.1%
Q30 Bases Count (bp)	7,798,359,715	7,251,470,951	752,491,533	7,231,616,645	7,920,864,107	7,576,240,376
Q30 Bases Ratio (%)	97.26%	97.27%	97.2%	96.85%	96.95%	96.92%
N Bases Count (bp)	433,618	367,761	400,689	362,907	438,470	400,689
N Bases Ratio (%)	0.01%	0.00%	0.00%	0.00%	0.01%	0.00%
GC Bases Count (bp)	4,261,930,436	3,997,774,689	412,985,256	4,012,761,140	4,300,578,456	415,666,980
GC Bases Ratio (%)	53.15%	53.62%	53.41%	53.74%	52.64%	53.72%
**Sample**	**CT1**	**CT2**	**CT3**	**C1**	**C2**	**C3**
Total Reads Count (#)	44,685,862	49,494,306	47,090,084	52,192,284	53,704,402	52,948,343
Total Bases Count (bp)	6,479,365,295	7,172,275,399	6,825,820,347	7,500,961,916	7,773,077,912	7,637,019,914
Average Read Length (bp)	145	144.91	144.96	143.72	144.74	144.23
Q10 Bases Count (bp)	6,461,773,661	7,151,595,460	6,806,684,561	7,480,328,311	7,754,647,673	7,617,487,992
Q10 Bases Ratio (%)	99.73%	99.71%	99.71%	99.72%	99.76%	99.76%
Q20 Bases Count (bp)	6,409,958,787	7,091,490,487	6,750,724,637	7,419,732,123	7,701,413,745	7,560,572,934
Q20 Bases Ratio (%)	98.93%	98.87%	98.97%	98.92%	99.08%	99.10%
Q30 Bases Count (bp)	6,245,530,856	6,900,969,339	6,573,250,098	7,226,834,821	7,532,886,563	7,379,860,692
Q30 Bases Ratio (%)	96.39%	96.22%	96.95%	96.35%	96.91%	96.61%
N Bases Count (bp)	312,621	367,599	340,110	381,956	386,130	384,043
N Bases Ratio (%)	0.00%	0.01%	0.00%	0.01%	0.00%	0.00%
GC Bases Count (bp)	3,524,789,488	3,844,896,391	3,684,842,940	4,075,942,614	4,132,313,249	4,104,127,932
GC Bases Ratio (%)	54.40%	53.61%	53.82%	54.34%	53.16%	54.20%

## Data Availability

The original contributions presented in this study are included in the article, is provided within the manuscript and Supplementary Information Files, further inquiries can be directed to the corresponding authors.

## Supplementary Materials

The following supporting information can be downloaded at: https://www.mdpi.com/article/10.3390/pharmaceutics17010066/s1.
