# Correction: Ren et al. Preparation of pH-Responsive Tanshinone IIA-Loaded Calcium Alginate Nanoparticles and Their Anticancer Mechanisms. *Pharmaceutics* 2025, *17*, 66

**DOI:** 10.3390/pharmaceutics18030294

**Published:** 2026-02-27

**Authors:** Tianying Ren, Jing Wang, Yingxin Ma, Yichen Huang, Somy Yoon, Lijun Mu, Ru Li, Xuekun Wang, Lina Zhang, Pan Li, Lusha Ji

**Affiliations:** 1 State Key Laboratory for Macromolecule Drugs and Large-Scale Manufacturing, College of Pharmacy, Wenzhou Medical University, Wenzhou 325035, China; dxmei_312@163.com; 2 State Key Laboratory for Macromolecule Drugs and Large-Scale Manufacturing, School of Pharmaceutical Sciences and Food Engineering, Liaocheng University, Liaocheng 252059, China; m13021553065@163.com (Y.M.); huangyichen200045@163.com (Y.H.); mlj15215354741@163.com (L.M.); liru0024@163.com (R.L.); xuekunwang0610@126.com (X.W.); 3 College of Pharmacy, Chonnam National University, Gwangju 61186, Republic of Korea; somyyoon@jnu.ac.kr; 4 Key Laboratory for Pediatrics of Integrated Traditional and Western Medicine, Liaocheng People’s Hospital, Liaocheng 252000, China; wangjing20083433@126.com; 5 College of Medicine, Liaocheng Vocational and Technical College, Liaocheng 252000, China; fangyan202109@163.com

## Correction of Reference

Our manuscript [1] cites a retracted paper (Tanshinone I attenuates the malignant biological properties of ovarian cancer by inducing apoptosis and autophagy via the inactivation of PI3K/AKT/mTOR pathway, https://onlinelibrary.wiley.com/doi/10.1111/cpr.13768). Since referencing a retracted paper could compromise the integrity of our work, I respectfully request the replacement of this citation and any associated discussion from our manuscript. The retracted paper was not foundational to our study. 

As revised, we have cited a new paper to show that Tan IIA exhibits an anticancer ability in many types of cancer in the Introduction.

After correction, Ref. [12] becomes the following:

Luo, H.; Vong, C.T.; Chen, H.; Gao, Y.; Lyu, P.; Qiu, L.; Zhao, M.; Liu, Q.; Cheng, Z.; Zou, J.; et al. Naturally occurring anti-cancer compounds: Shining from Chinese herbal medicine. *Chin Med.* **2019**, *14*, 48. https://doi.org/10.1186/s13020-019-0270-9

The overall numbering and order of the references remain unchanged. The authors state that the scientific conclusions are unaffected. This correction was approved by the Academic Editor. The original publication has also been updated.
