# Supplementary figures and images for: Preparation of pH-Responsive Tanshinone IIA-Loaded Calcium Alginate Nanoparticles and Their Anticancer Mechanisms
Source: Pharmaceutics. 2025 Jan 6;17(1):66. doi: 10.3390/pharmaceutics17010066 (PMC11768977; doi:10.3390/pharmaceutics17010066)

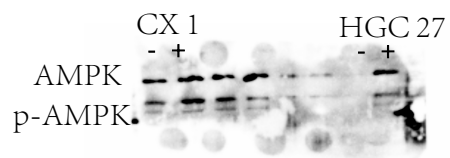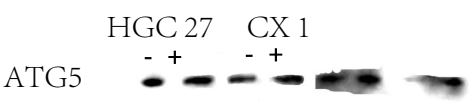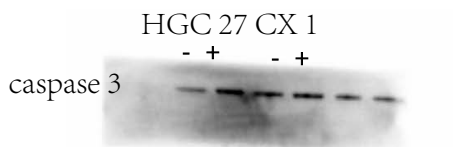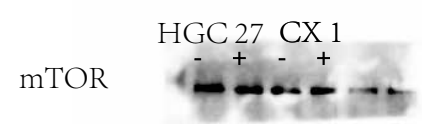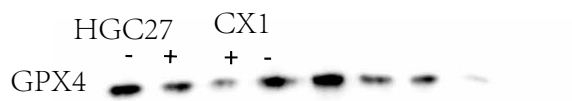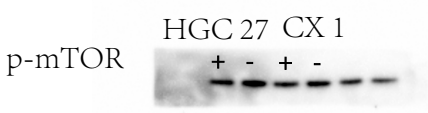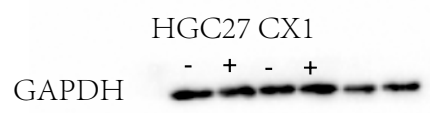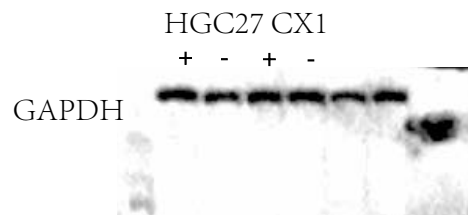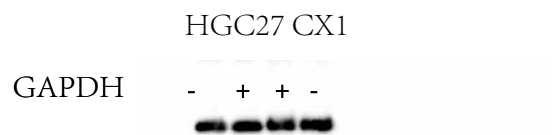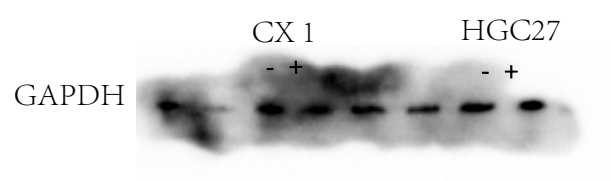

Supplement: Supplementary file 1 [file pharmaceutics-17-00066-s001.zip › pharmaceutics-3296260-supplementary.pdf]
